# Mass Spectrometric Analysis of Differentially Expressed Proteins in an Endangered Medicinal Herb, *Picrorhiza kurroa*

**DOI:** 10.1155/2014/326405

**Published:** 2014-05-04

**Authors:** Amit Sud, Rajinder Singh Chauhan, Chanderdeep Tandon

**Affiliations:** Department of Biotechnology and Bioinformatics, Jaypee University of Information Technology, Waknaghat, Solan 173234, India

## Abstract

*Picrorhiza kurroa* grown in the Northwestern Himalayan region is used in various herbal formulations but extensive harvesting of this plant has led it to near extinction. The active constituents responsible for the medicinal properties of *P. kurroa* have been identified as picroside-I and picroside-II which are present in a particular ratio (1 : 1.5) in herbal formulations like Picroliv. The biosynthetic pathway of picrosides has been partially deciphered till date and needs to be elucidated completely. Review of literature revealed that no information is available as of today on the proteome analysis of *Picrorhiza kurroa* w.r.t. picroside-II biosynthesis. Therefore, with the aim of identifying proteins associated with picroside biosynthesis in *Picrorhiza kurroa*, differential protein expression was studied under picroside accumulating versus nonaccumulating conditions using SDS-PAGE. A total of 19 differentially expressed proteins were identified using MALDI-TOF/TOF MS followed by MASCOT search. Proteins involved in diverse functions were identified amongst which the most important proteins were glyceraldehyde-3-phosphate dehydrogenase, 1-aminocyclopropane-1-carboxylate oxidase, photosystem I reaction centre subunit V, 2-oxoglutarate ferrous-dependent oxygenase and putative cytochrome P450 superfamily protein because of their role in picroside biosynthesis. These identified proteins provide an insight and a basic platform for thorough understanding of biosynthesis of secondary metabolites and various other physiological processes of *P. kurroa*.

## 1. Introduction

Plants have become an important part of our daily lifestyle. For several years, intensive studies have been carried out on various plant constituents and their nutrition value [1]. Plants synthesize a variety of metabolites which can be classified into two categories, namely, primary metabolites which are involved in essential metabolic processes of the plant and secondary metabolites which are not involved in the fundamental life processes of plant but in a variety of other roles which help plants in their survival and reproduction [1–4].

The medicinal properties of various medicinal plants can be attributed to these secondary metabolites. It has been generally observed that a particular medicinal property is inherent to a specific plant species or groups which is in congruence with the fact that taxonomically distinct plants possess different combination of secondary metabolites [5].

One such medicinal plant is* Picrorhiza kurroa* which is a perennial medicinal herb belonging to family Scrophulariaceae found in northwestern Himalayas at an altitude from 3000 m to 4300 m [6–8]. It has been widely used in the ancient Ayurvedic system for treatment of various disorders like liver diseases, dyspepsia, chronic diarrhoea, and upper respiratory ailments [6]. In modern system of medicine, it is widely used as a hepatoprotective [6–11], anticarcinogenic [12], antioxidant [7, 8, 11, 13, 14], antiallergic [8], antiasthmatic [8, 11, 15], immunomodulatory [8, 11, 14], superoxide scavenging [10], antidiabetic [16], and immunostimulant [8, 17].

Research on* P. kurroa* extracts has revealed its potential role in treating hepatic injuries induced in rats by various agents such as ethanol [8, 18], thioacetamide, galactosamine, and carbon tetrachloride [8, 19].

These pharmacological activities of* P. kurroa* are due to the presence of novel monoterpene derived iridoid glycosides such as picroside-I, picroside-II along with picroside-III, picroside-IV, apocynin, androsin, catechol, kutkoside, verminoside, and specioside [6–8].

*P. kurroa* has been used in various herbal formulations like Picroliv, Livokin, Picrolax, Livomap, Tefroliv, Katuki, Arogya, Kutaki, and so forth [7, 8]. The requirement of a particular concentration and composition of desired chemical constituents in a herbal drug formulation has been emphasized by Picroliv, which is a herbal formulation from* P. kurroa* and reported to contain a definite ratio (1 : 1.5) of picroside-I and picroside-II [20]. The upsurge in market demand, inadequate cultivation, and uncontrolled collection from the wild has resulted in declaring* P. kurroa* as a critically endangered species [7].

The two major medicinal components of* P. kurroa*, picroside-I (P-I) and picroside-II (P-II), show differential accumulation. P-I has been found to be differentially produced in shoots, while P-II has been found to occur differentially in stolons or roots [8, 11, 18].

Recently, the pathway for the production of picrosides has been proposed. Picrosides are derived from geranyl diphosphate (GDP) using the isoprenoid biosynthetic pathway [7]. The search for whether this GPP was derived from the mevalonate (MVA) pathway or the 2-*C*-methyl-D-erythritol 4-phosphate (MEP) pathway resulted in the identification of several upregulated genes of both MVA and MEP pathways indicating that both pathways contributed in the formation of GPP [7, 18].

All picrosides are formed from the esterification of catalpol derived from iridoid biosynthetic pathway with various aromatic acids obtained from phenylpropanoid pathway [7].

Although the metabolites of the pathway have been deciphered, the proteins involved in their biosynthesis still need to be elucidated. According to a proposed strategy, metabolites, proteins, and transcriptional profiling under two physiological states (e.g., metabolites accumulating versus nonaccumulating) can provide a novel approach for pathway elucidation in plants [8, 20].

Although transcriptomics analysis can provide a great wealth of knowledge about various biological processes, it is incomplete without proteome analysis which aids in improving the understanding of events occurring inside a cell [8]. As the central dogma dictates the transcription of DNA to mRNA and the translation of mRNA to proteins, one would expect to find correlation between mRNA and protein abundances. But this is not true; research has found the correlation between mRNA and protein to be poor [8, 21–23].

Three reasons have been presumed for this poor correlation: (a) significant difference in the half lives of proteins, (b) inability to get a clear picture because of significant amount of error and noise in both protein and mRNA experiments, and (c) various complicated posttranslational modifications [23]. It has been observed that DNA sequence and mRNA expression studies fail to provide information regarding protein posttranslational modification, structure, and protein-protein interactions. For performing various functions, almost all proteins undergo posttranslational modification; hence, it becomes essential to analyse the protein content so as to get a better understanding of the various physiological processes [8, 24].

Another important reason which necessitates the study of* P. kurroa* proteins is that proteomic studies till date have been carried out primarily in model plants such as* Arabidopsis thaliana*,* Oryza sativa* (rice),* Populus trichocarpa* (black cottonwood), and* Vitis vinifera* (grape vine) for which fully sequenced genomes are available. Very few studies have been conducted in relation to the biosynthesis of secondary metabolites in medicinal plants especially with the focus of identifying new enzymes involved in secondary metabolism [25].

Review of literature reveals that no proteomic data is available as of today associated with the biosynthesis of picroside-II in the stolons of* P. kurroa*. Hence this preliminary study was carried out with the objective of identifying proteins for the first time related to the biosynthesis of picroside-II in the stolon of* Picrorhiza kurroa* using differential proteomics approach between two differential conditions of metabolite accumulation and metabolite nonaccumulation.

## 2. Material and Methods

### 2.1. Plant Material

For differential proteomic study, roots were obtained from* P. kurroa* plants maintained by tissue culture at 15°C and stolon samples were taken from* P. kurroa* plants obtained from Sairopa (4,500 m altitude, 31°38′–31°54′N, and 77°20′–77°45′E) with respect to differential picroside-II content. Both of the samples were obtained from the same strain of* P. kurroa* deposited at Himalayan Forest Research Institute, Shimla, India, with Herbarium Accession no. 0670. These samples were selected on the basis of ~10 times higher picroside-II content (as determined by HPLC) in Sairopa plants (10.4 mg/g fresh weight) as compared to roots of* P. kurroa* grown at 15°C (0 mg/g fresh weight).

### 2.2. Picroside-II Quantification

Picroside-II content was estimated using high performance liquid chromatography (HPLC) analysis method developed by Sood and Chauhan [11]. P-II content in 15°C roots has already been experimentally proven to be 0 mg/g fresh weight [18]. For quantification of P-II in the stolon samples, they were dissolved in methanol after being grounded into a fine powder using liquid nitrogen. The filtered extract was then diluted 10 times and analysed using reverse phase (HPLC Waters 515) through C18 (5 *μ*m) 4.6 × 250 mm Waters Symmetry Column using PDA detectors (Waters 2996). Two solvent systems were used for running the test samples, that is, solvent A (0.05% trifluoroacetic acid) and solvent B (1 : 1 methanol/acetonitrile mixture. Solvents A and B were used in the ratio 70 : 30 (v/v). The column was eluted in isocratic mode with flow rate of 1.0 mL/min. P-II was detected at 270 nm. The cycle time of analysis was 30 minutes at 30°C. The compounds were identified on the basis of retention time and comparison of UV spectra with the authentic standard from ChromaDex, Inc.

### 2.3. Protein Extraction

The samples were excised and washed with sterile water. These washed and dried samples were frozen in liquid nitrogen and grounded in a precooled pestle and mortar to obtain a fine powder. This fine powder was then suspended in 10% (w/v) trichloroacetic acid (TCA) in 100% (v/v) acetone containing 0.07% (w/v) dithiothreitol (DTT). For complete precipitation, samples were incubated overnight at −20°C, followed by centrifugation at 15,557 ×g for 45 minutes. The pellets were resuspended in 100% (v/v) acetone containing 0.07% DTT for 1 hour, followed by centrifugation at 15,557 ×g for 45 minutes. This step was repeated thrice to completely remove any residual TCA. The pellet was then air dried to remove acetone and resuspended in lysis buffer containing 7 M urea, 2 M thiourea, 2% (w/v) 3-[(3-cholamidopropyl) dimethylammonio]-1-propanesulfonate (CHAPS), 1% (w/v) DTT, 2% biolyte pH 3–10, and protease inhibitor cocktail. The samples were then sonicated in a water bath maintained at 20°C for 30 minutes followed by gentle stirring at room temperature for 4 hr. The samples were centrifuged at 15,557 ×g for 45 minutes and supernatant obtained was transferred to a new tube for further centrifugation at 15,557 ×g for 45 minutes to remove any residual precipitate. The clear supernatant obtained was divided into aliquots and stored at −80°C. Protein concentration was estimated using the Bio-Rad RC DC protein estimation kit.

### 2.4. Sodium Dodecyl Sulphate: Polyacrylamide Gel Electrophoresis (SDS-PAGE) Analysis

For SDS-PAGE analysis, the required amount of the samples was precipitated by adding 100% (v/v) acetone containing 0.07% DTT in 1 : 4 ratio. The samples were kept at −20°C for at least 2 hours, followed by centrifugation at 20,000 ×g for 15 minutes. The pellet obtained was air dried at room temperature and resuspended in 2x Laemmli buffer containing 0.5 mM Tris-HCl (pH 6.8), 25% glycerol, 1% bromophenol blue, and 10% SDS. The samples were loaded onto a discontinuous gel system containing 12% resolving gel and 5% stacking gel. Separation was carried out using Bio-Rad PROTEAN II xi cell at 16 mA for first 30 minutes, followed by 24 mA till dye front reached bottom of the gel. The gels were visualized by silver staining. SDS-PAGE gel separations were repeated for a total of 3 times.

### 2.5. Image Acquisition and Analysis

The SDS-PAGE gels were scanned using Bio-Rad GS-800 calibrated densitometer at a resolution of 36.3 × 36.3 microns. All image analysis and densitometry studies were performed using Bio-Rad's Quantity One software.

### 2.6. MALDI TOF/TOF MS Analysis

The differentially expressed bands identified after the image analysis were excised and cut into small pieces of approximately 1 mm followed by destaining of the pieces in a freshly prepared 1 : 1 (v/v) mixture of potassium ferricyanide and sodium thiosulphate. The gel pieces were then sequentially incubated with reducing and alkylating reagents and with modified trypsin (Sigma). Peptides were eluted and reextracted in 50% trifluoroacetic acid (TFA) containing 0.1% acetonitrile (ACN). The samples were purified using ZipTip and mixed with *α*-cyano-4-hydroxycinnamic acid (4-*HCCA*) matrix in 1 : 1 ratio followed by plating onto a MALDI plate. After air drying, the plate was analysed using MALDI TOF/TOF ultraflex III instrument and further analysis was done with flex analysis software for obtaining the peptide mass fingerprint.

### 2.7. MASCOT Protein Identification

The data obtained from MALDI-TOF/TOF MS analysis was used to identify proteins using the MASCOT protein database search engine maintained at http://www.matrixscience.com. Peptides were assumed to be monoisotopic, carbamidomethylated at cysteine residues, and oxidized at methionine residues. Only 1 maximal cleavage was allowed for peptide matching. Swissprot and NCBInr databases were searched with Viridiplantae as the preferred taxonomy. Proteins with probability based MOWSE scores exceeding their threshold (*P* < 0.05) were considered to be positively identified.

## 3. Results and Discussion

### 3.1. Results

#### 3.1.1. HPLC Analysis

**Figure 1:**
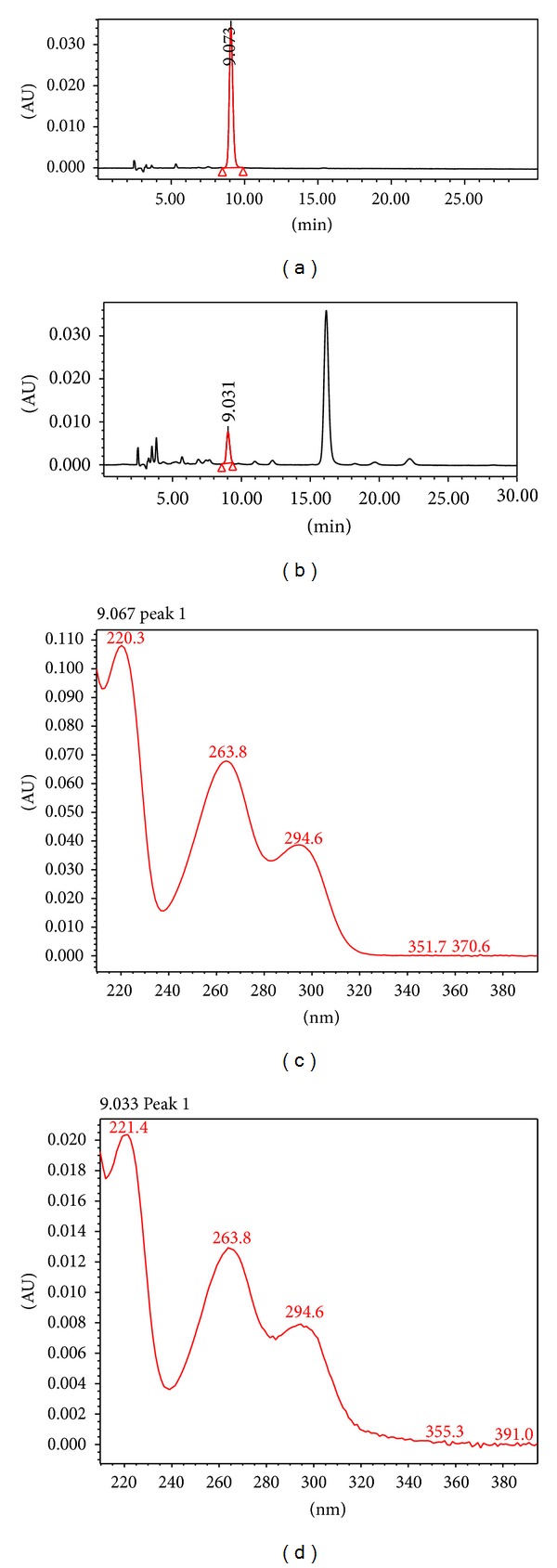
HPLC analysis for quantification of P-II content in Sairopa stolons. (a) HPLC chromatogram of P-II standard, (b) HPLC chromatogram of Sairopa stolon sample, (c) UV spectra of P-II standard, and (d) UV spectra of Sairopa stolon sample.

The estimation of picroside-II content using HPLC identified approximately 10 times more P-II in case of Sairopa stolons as compared to 15°C roots. The P-II content in Sairopa stolons was estimated to be 10.4 mg/g fresh weight of stolons. As far as 15°C roots are concerned, the P-II content was estimated to be 0 mg/g fresh weight of 15°C roots [18].  Figure 1 shows the presence of picroside-II in Sairopa stolons which was validated by comparing the chromatogram and the UV spectra of the Sairopa sample with that of P-II standard.

#### 3.1.2. SDS-PAGE Analysis

**Figure 2:**
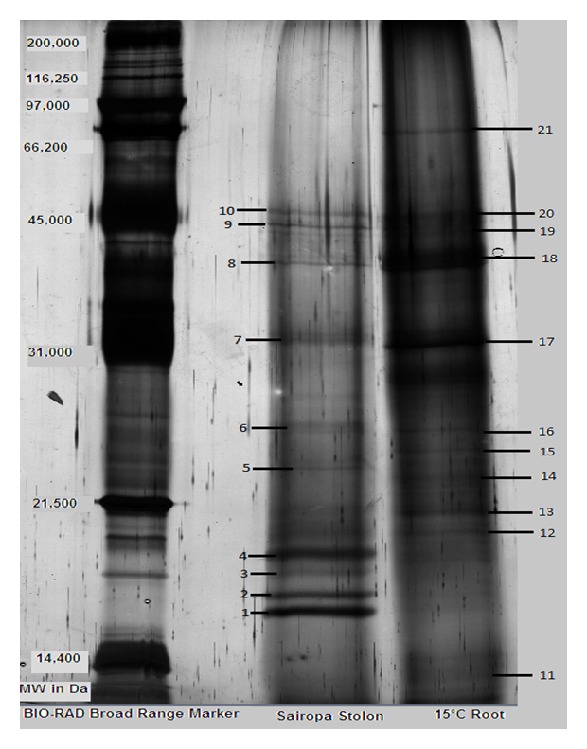
SDS PAGE profile of Sairopa stolon and 15°C roots with marked bands that were excised for MALDI-TOF/TOF MS analysis based on their differential expression.

**Figure 3:**
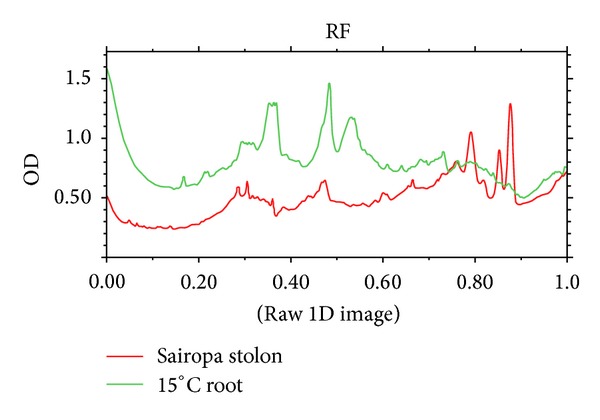
Densitogram analysis of SDS-PAGE gel using Bio-Rad's Quantity One software showing the relative optical densities and relative front of various bands. The red line represents Sairopa stolon samples and the green line represents 15°C root samples. The different peaks indicate the different bands in the samples and their heights correspond to the level of expression.

Differential protein expression studies revealed a total of 29 bands in Sairopa and 26 bands in 15°C root samples (Figure 2). Densitometry analysis (Figure 3) of the gel using Bio-Rad's Quantity One software identified a total of 21 differentially expressed proteins, out of which 10 proteins were differentially expressed in Sairopa stolons, while 11 were differentially expressed in 15°C roots. Gel analysis showed that most of the proteins were concentrated between a molecular weight range from 15 kDa to 45 kDa.

#### 3.1.3. Identification of Differentially Expressed Proteins

**Figure 4:**
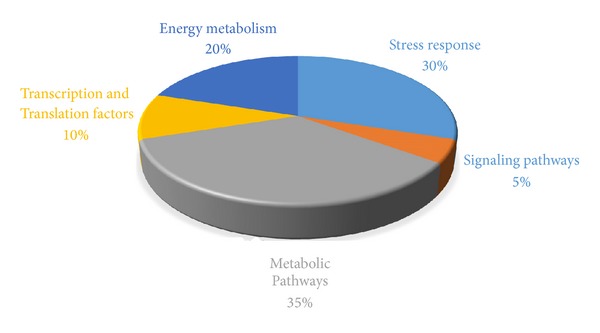
Putative functional classification of differentially expressed proteins.

Mass by charge ratios of 21 differentially expressed proteins obtained from MALDI-TOF/TOF MS were used to search the MASCOT database. Out of 21, 19 differentially expressed proteins were identified.  Table 1 shows the differentially expressed proteins identified after MASCOT analysis. These proteins were involved in stress response, signalling pathways, metabolic pathway, transcription, and energy metabolism. The functional distribution of proteins is represented in Figure 4. The majority of proteins were found to be involved in stress response and metabolic pathways.

Proteins involved in (a) stress response include methionine sulfoxide reductase, peptidyl-prolyl* cis-trans* isomerase, DnaJ homolog subfamily C, glyceraldehyde-3-phosphate dehydrogenase, 1-aminocyclopropane-1-carboxylate oxidase, and chaperone protein DnaJ; (b) signaling pathways include Rab GTPase; (c) metabolic pathway includes glyceraldehyde-3-phosphate dehydrogenase, 1-acyl-sn-glycerol-3-phosphate acyltransferase 2, 2-oxoglutarate ferrous-dependent oxygenase, UbiE/COQ5 methyltransferase, putative cytochrome P450 superfamily protein, adenylate isopentenyltransferase, and 3-ketoacyl-CoA synthase 11; (d) transcription and translation include predicted ethylene-responsive transcription factor WIN1 and 40S ribosomal protein; and (e) energy metabolism includes ferredoxin, photosystem 1 reaction centre subunit V, NADH dehydrogenase subunit F, and mitochondrial carrier protein.

### 3.2. Discussion

In this study, an attempt was made to identify differentially expressed proteins in* Picrorhiza kurroa* samples grown under different conditions of picroside accumulation and nonaccumulation. The proteomic analysis of medicinal plants in the absence of fully sequenced and annotated genomes can allow exploration and investigation of physiological pathways related to metabolism, defense, signaling, and energy metabolism of these medicinal plants [25].

It has been experimentally proven that picroside-II content in Sairopa stolon is about ~10 times higher as compared to 15°C root samples. The estimation of P-II content in different tissues helped in the identification of differential conditions for the biosynthesis of P-II. The higher content of P-II in case of Sairopa stolons can be attributed to various climatic factors such as light, temperature, altitude, and UV [18]. When same amount of protein (15 *μ*g) was loaded onto the gel, differentially expressed proteins were identified in Sairopa stolon. This is in congruence with the fact that the amount of picroside-II in Sairopa sample is 10.4 mg/g fresh weight as compared to 0 mg/g fresh weight in 15°C root samples as determined by HPLC.

#### 3.2.1. Stress Related Proteins

Sairopa samples obtained from high altitudes and grown in green house were under more stress as compared to 15°C samples grown using tissue culture. This resulted in a number of stress related proteins being uniquely expressed in Sairopa sample. Methionine sulfoxide reductase catalyses the reduction of methionine sulfoxide to methionine which is oxidised to methionine sulfoxide under oxidation conditions. This results in a change in protein hydrophobicity and its folding ultimately affecting its catalytic function [26–29]. For example, a heat shock protein, Hsp21 loses its chaperone activity when methionine residues are oxidised. The action of methionine sulfoxide reductase helps in attaining a fully active enzyme by reduction of these oxidised methionine residues using thioredoxin as the reductant [26, 30]. Peptidyl-prolyl* cis-trans* isomerases also known as conformases or rotamases are involved in protein folding because of their ability to catalyse slow steps in the initial folding/rearrangement of proteins [31, 32]. Peptidyl-prolyl* cis-trans* isomerase functions to prevent or reverse protein aggregation resulting from stress conditions [32]. DnaJ is part of the DnaK-DnaJ chaperone system which is centrally involved in heat stress response in response to destabilizations which cause protein misfolding [33]. Although DnaJ can act as a chaperone on its own, it generally functions as a cochaperone with DnaK [34]. Glyceraldehyde-3-phosphate dehydrogenase catalyzes the oxidation of triose phosphates during glycolysis. In addition to this role, it is also involved in stress conditions [35, 36] where it has been shown to interact with the plasma membrane-associated phospholipase D to transduce the ROS hydrogen peroxide signal [36]. It is assumed that it acts as a part of signaling pathway to increase malate-valve capacity and the effect of other protective systems [35]. 1-Aminocyclopropane-1-carboxylate oxidase is involved in ethylene biosynthesis in response to various biotic and abiotic stress conditions [37]. It catalyses the oxidation of 1-aminocyclopropane-1-carboxylate (ACC) to ethylene [38]. Previous studies showed that changes in monoterpene concentration are generally related to the rate of ethylene production; that is, with high rates of ethylene production monoterpene concentrations were also found to be increased [39]. Therefore, an overexpression of this enzyme indicated a possibility of its involvement in picroside biosynthesis. Chaperone DnaJ-like protein is involved in protein folding and assembly. Different DnaJ-like proteins interact with specific Hsp70s forming pairs adapted to each other and function as chaperone system protecting plants against various stress conditions [40].

#### 3.2.2. Signaling Pathways Related Proteins

Plants require vesicular transportation for various specialized phenomena and common housekeeping events. Rab GTPase is the key player involved in vesicular transport. They act as molecular switches controlling the fusion of vesicles with target membranes via transition between GTP and GTP-bound forms [41].

#### 3.2.3. Metabolic Pathway Related Proteins

**Figure 5:**
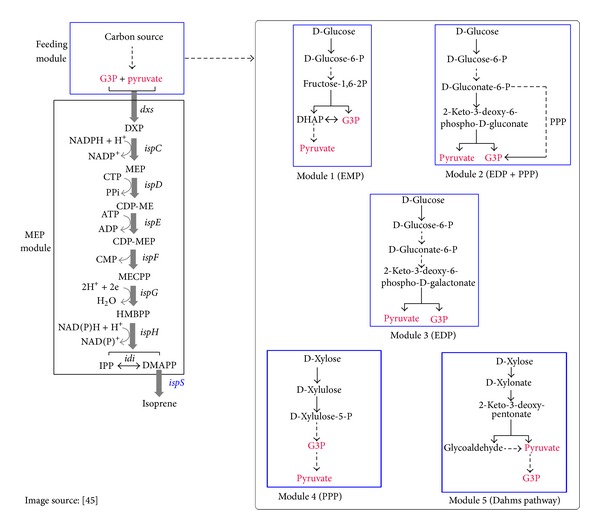
This image shows the connecting link of MEP pathway to other pathways for supply of glyceraldehyde-3-phosphate and pyruvate, the two starting molecules of MEP pathway. Module 1, that is, glycolysis, is of interest as the overexpressed enzyme glyceraldehyde-3-phosphate dehydrogenase is involved in this pathway. Gene symbols and the enzymes they encode:* dxs*: DXP synthase;* ispC*: DXP reductoisomerase;* ispD*: DXP-ME synthase;* ispE*: CDP-ME kinase;* ispF*: MECPP synthase;* ispG*: HMBPP synthase;* ispH*: HMBPP reductase;* idi*: IPP isomerase;* ispS*: isoprene. Pathway intermediates: G3P: glyceraldehyde-3-phosphate; DXP: 1-deoxy-D-xylulose 5-phosphate; MEP: 2-*C*-methyl-D-erythritol 4-phosphate; CDP-ME: 4-diphosphocytidyl-2-*C*-methyl-D-erythritol; CDP-MEP: 4-diphosphocytidyl-2-*C*-methyl-D-erythritol 2-phosphate; MECPP: 2-*C*-methyl-D-erythritol 2,4-cyclopyrophosphate; HMBPP: 1-hydroxy-2-methyl-2-(*E*)-butenyl 4-pyrophosphate; IPP: isopentenyl pyrophosphate; DMAPP: dimethylallyl pyrophosphate; DHAP: dihydroxyacetone 3-phosphate.

Glyceraldehyde-3-phosphate dehydrogenase is associated with glycolysis where it catalyses the reversible reaction of converting glyceraldehyde-3-phosphate to 1,3-bisphosphoglycerate [36, 41]. This enzyme is involved in energy production and siphoning of various intermediates for cellular metabolism [41]. Overexpression of this enzyme leads to production of more pyruvate which is one of the starting molecules of MEP pathway involved in the biosynthesis of picrosides (Figure 5) [42].

Phospholipids are responsible for maintaining the epidermal permeability barrier. This barrier prevents transcutaneous water loss helping in plant survival. 1-Acyl-sn-glycerol-3-phosphate acyltransferase 2 is the key enzyme involved in the biosynthesis of phospholipids and triglycerides. This enzyme causes acylation of lysophospholipids to phosphatidic acid which is the major precursor of all phospholipids/triglycerides [43]. 2-Oxoglutarate ferrous-dependent oxygenase is a superfamily of enzymes that are known to catalyse various reactions like hydroxylations, desaturations, and oxidative ring closures [44]. They are involved in posttranslational modification of collagen and in biosynthesis of both primary and secondary metabolites [44] including flavonoid biosynthesis which are a kind of secondary metabolites derived from phenylalanine and acetate metabolism. 2-Oxoglutarate ferrous-dependent oxygenase catalyses different steps within the same pathway due to the fact that flexibility in metal coordination chemistry suggests its suitability for new and unusual reactions [44]. UbiE/COQ5 methyltransferase belongs to family of methyltransferases which participate in the biosynthesis of menaquinone and ubiquinone. Ubiquinone is involved in the respiratory chain where it transfers electron from complex I (or complex II) to complex III. Ubiquinone has been explored for roles other than in electron transfer such as its role in/as antioxidant, disulphide bond formation, and extension of lifespan due to lack of ubiquinone [45]. All of these functions suggest its importance in survival. Cytochrome P450 enzymes are involved in various biosynthetic and detoxification pathways. In biosynthetic pathways, these enzymes have played tremendous role in biosynthesis of lignin intermediates, sterols, terpenes, flavonoids, isoflavanoids, furanocoumarins, and other secondary metabolites [46]. Cytochrome P450 dependent monooxygenases have been found to increase the structural diversity of terpenoids [47]. Adenylate isopentenyl transferase is involved in cytokinin biosynthesis where it catalyses the transfer of an isopentenyl group from dimethylallyl diphosphate (DMAPP) to N6 amino group of adenosine phosphate to produce isopentenyl adenosine phosphates [48, 49] which are then converted to isopentenyladenine and* trans*-zeatin [48]. These cytokinins are involved in plant growth and development [49]. 3-Ketoacyl-CoA synthase 11 is involved in the biosynthesis of cuticular wax and suberin [50]. Cuticle present on plant surfaces acts as the first line of defense against pathogens, phytophagous insects, and environmental stresses such as drought, UV damage, and frost. Overexpression of this protein confers protection to* Picrorhiza kurroa*. Differential expression of all the proteins in this category helps in plant survival and in the formation of secondary metabolites. Although the pathway for picrosides biosynthesis is yet to be fully deciphered, the overexpression of these enzymes adds significantly to the available information.

#### 3.2.4. Transcriptional and Translational Factors Related Proteins

Regulating genes at transcription level serves as one of the most important points of regulation in biological processes. Ethylene responsive transcription factor WIN1 has been shown to be related to plant development, defense response, and stress signaling pathways enabling plants to adjust to their adverse surroundings [51]. It promotes cuticle formation by inducing the expression of enzymes involved in wax biosynthesis. It provides protection against drought resistance [52]. 40S ribosomal protein S13-1 is involved in translation of mRNA to proteins which may help plants to synthesize new proteins or replace damaged proteins to help plants cope with various stress conditions. Overexpression of this protein indicated that stressed plant is undergoing heavy translation.

#### 3.2.5. Energy Metabolism Related Proteins

Ferredoxin belongs to family of oxidoreductases which use iron-sulfur proteins as electron donors and NAD^+^ or NADP^+^ as electron acceptors. These function primarily in photosynthesis where they transfer electrons from photoreduced photosystem I to ferredoxin NADP(+) reductase in which NADPH is produced for CO_2_ assimilation [53]. Ferredoxin can also function in removing excessive reducing power and preventing uncontrolled overreduced states that are common in stroma under stress conditions [54]. Photosystem I reaction centre subunit V of the photosystem I is an integral membrane protein [55]. It is involved in stabilizing the binding of peripheral antenna and regulation of photosystem I [56]. Photosystem I functions to produce NADPH necessary for the reduction of CO_2_ in the Calvin-Benson-Bassham cycle. This cycle has been previously shown to be linked to MEP pathway for the synthesis of picrosides [8, 42]. Photosystem I is involved in cyclic synthesis of ATP from light generating large amounts of ATP for sustaining various metabolic and physiological processes [57]. NADH dehydrogenase subunit F is a part of NADH dehydrogenase. NADH dehydrogenase allows electron transport to continue even when membrane potential is high, thus uncoupling electron transport from ATP synthesis. Removal of intermediates from the citric acid cycle, as what happens during secondary metabolite synthesis, requires NAD^+^ to be recycled at a higher rate than coupled transport allows. This problem is solved by NADH dehydrogenase catalysed recycling of NAD^+^ in mitochondrial matrix [58]. Mitochondria play an important role in respiration and energy production and are involved in several plant metabolic pathways. Mitochondrial carrier family proteins connect the internal metabolism with that of the cells surrounding allowing exchange of ATP, di- and tricarbonic acids basic amino acids, carnitine, S-adenosylmethionine, phosphate, reducing power, and so forth, from and to the mitochondria [59].

## 4. Conclusions

*Picrorhiza kurroa* is associated with innumerable medicinal properties, most potent being the ability to treat various liver disorders. Herbal formulations meant for treating hepatic disorders use a particular ratio of picroside-I and picroside-II as the main active components. Therefore, it becomes all the more important to study their biosynthesis so as to understand their biosynthesis within* P. kurroa* which is partially known till date. Studying difference in proteomes of samples corresponding to metabolite accumulation and nonaccumulation can help in elucidating important proteins involved directly or indirectly in the biosynthesis of these potent molecules. Differential proteomic analysis can aid in profiling altered proteins enabling better understanding of various physiological processes occurring in plants. Here, differential proteome analysis using SDS-PAGE combined with mass spectrometry based protein identification revealed altered proteins belonging to several functional categories like stress response, signaling pathways, metabolic pathways, transcription and translation factors, and energy metabolism. As this is the first report on the analysis of proteins from stolon and roots of different* Picrorhiza kurroa* samples grown under metabolite accumulating and nonaccumulating conditions, all the identified proteins will contribute in understanding the various physiological processes of this plant. Out of the 21 differentially expressed proteins, the proteins of notable importance were identified as glyceraldehyde-3-phosphate dehydrogenase, 1-aminocyclopropane-1-carboxylate oxidase, photosystem I reaction centre subunit V, 2-oxoglutarate ferrous-dependent oxygenase, and putative cytochrome P450 superfamily protein because of their role in picroside biosynthesis. Hence, information resulting from this study can provide an important platform for carrying further the research using advance proteomics techniques such as 2D gel electrophoresis and shotgun proteomics to identify more proteins enabling a thorough understanding of the plants physiological processes. Identification of all the proteins involved in the biosynthesis of picrosides would allow the complete pathway to be deciphered including the identification of rate limiting enzyme which can be enhanced to increase the picroside yield. MEP and MVA pathways involved in picroside biosynthesis are expressed in several other organisms like plants and microorganisms. Metabolic engineering of this pathway into these organisms can reduce the overexploitation of* Picrorhiza kurroa* for obtaining picrosides and thus preventing it from getting extinct.

## Figures and Tables

**Table 1:** Differentially expressed proteins identified from MALDI-TOF/TOF MS data using MASCOT search engine.

Band number	Protein identified	Accession number	M.W. (Da)	% Seq. Coverage	SCORE	*e*-value	Functions
1	Methionine sulfoxide reductase	XP_003594067	15,446	29	54	0.062	Methionine sulfoxides can be reduced back to the methionines by a thioredoxin-dependent enzyme, peptide methionine sulfoxide reductase (Msr), providing cells with a mechanism to repair proteins damaged by reactive oxygen species rather than having them degraded and then resynthesizing them *de novo*.

2	Ferredoxin-6	NP_001147617	15,334	14	60	0.045	It uses iron-sulfur proteins as electron donors and NAD^+^ or NADP^+^ as electron acceptors. These function primarily in photosynthesis where they transfer electrons from photoreduced photosystem I to ferredoxin NADP (+) reductase in which NADPH is produced for CO_2_ assimilation.

3	40S ribosomal protein S13-1	Q69UI2	17,105	46	59	0.048	Is involved in protein translation.

4	Peptidyl-prolyl *cis-trans* isomerase	P35627	18,320	27	55	0.058	Peptidyl-prolyl *cis-tran*s isomerases have been termed conformases or rotamases because they catalyse slow steps in the initial folding/rearrangement of proteins.

5	Rab GTPase B1C isoform 2	EOY29043	21,168	43	52	0.069	Key players of vesicular transport. They act as molecular switches regulating the fusion of vesicles with target membranes through the conformational change between GTP- and GDP-bound forms.

6	DnaJ homolog subfamily C member 28-like	XP_004497574	22,904	40	54	0.062	Play central roles in the heat stress. Act as cochaperone along with DnaK in the DnaK-DnaJ system. DnaJ is able to act as a chaperone on its own, but its main role is as cochaperone interacting with DnaK.

7	Glyceraldehyde-3-phosphate dehydrogenase	ABD37966	34,541	20	68	0.026	Enzyme of the glycolytic pathway responsible for the conversion of glyceraldehyde 3-phosphate to D glycerate-1, 3,-bisphosphate. It also acts as reversible metabolic switch under oxidative stress.

8	1-Aminocyclopropane-1-carboxylate oxidase	XP_002532366	38,381	25	56	0.056	Plays an important role in regulation of ethylene formation in plants in response to various stress conditions.

10	1-Acyl-sn-glycerol-3-phosphate acyltransferase 2-like	XP_004136588	43,818	17	61	0.042	It is a key enzyme of phospholipid and triglyceride biosynthesis. Several lines of evidence suggest that AGPAT should play a key role in the synthesis of phospholipids/triglycerides required for permeability barrier homeostasis.

11	Photosystem I reaction center subunit V, chloroplast precursor	XP_002949489	13,343	30	72	0.018	Encodes subunit G of photosystem I, an 11 kDa membrane protein that plays an important role in electron transport between plastocyanin and PSI and is involved in the stability of the PSI complex. It also takes part in cyclic photophosphorylation resulting in ATP production without NADPH generation.

12	2-oxoglutarate ferrous-dependent oxygenase	ADY80557	20,760	27	52	0.068	The 2-oxoglutarate (2-OG) and ferrous iron dependent oxygenases are a superfamily of enzymes that catalyse a wide range of reactions including hydroxylations, desaturations, and oxidative ring closures. 2-OG oxygenases catalyse reactions in the posttranslational modification of collagens and in the biosynthesis of both primary and secondary metabolites. They have also been identified as catalysing steps in the biosynthesis of plant signalling molecules including ethylene and the gibberellins.

13	Predicted: ethylene-responsive transcription factor WIN1-like	XP_004152351	21,950	33	57	0.053	Promotes cuticle formation by inducing the expression of enzymes involved in wax biosynthesis. Confers drought resistance. Acts as a transcriptional activator. Binds to the GCC-box pathogenesis-related promoter element.

14	UbiE/COQ5 methyltransferase	XP_003078783	24,603	36	66	0.033	Involved in ubiquinone biosynthesis.

15	Putative cytochrome P450 superfamily protein	AFW88279	23,550	25	64	0.038	Cytochrome P450 (CYP) enzymes are a superfamily of monooxygenases that are found in all kingdoms of life and which show extraordinary diversity in their reaction chemistry. Important for the biosynthesis of several compounds, such as hormones, defensive compounds, and fatty acid conjugates.

16	NADH dehydrogenase subunit F	ABB93079	27,880	28	52	0.068	Catalyses the transfer of electrons from NADH to coenzyme Q (CoQ). It is the “entry enzyme” of oxidative phosphorylation in the mitochondria.

17	Mitochondrial carrier family	XP_002502691	29,524	36	59	0.048	Solute carriers in the inner mitochondrial membrane which connect the internal metabolism with that of the surrounding cell. It is known to catalyse the specific transport of various substrates, such as nucleotides, amino acids, dicarboxylates, cofactors, phosphate, or H^+^.

18	Adenylate isopentenyl transferase 8	Q9LJL4	37,583	23	67	0.029	Involved in cytokinin biosynthesis. Catalyzes the transfer of an isopentenyl group from dimethylallyl diphosphate (DMAPP) to ATP and ADP.

20	Chaperone protein DnaJ-like	XP_002274349	48,696	13	55	0.058	Members of the DnaJ-like protein family act as chaperones through direct interaction with different Hsp70 acting in protein pairs that appear to be specifically adapted to each other.

21	3-Ketoacyl-CoA synthase 11-like isoform 1	XP_004251584	57,764	12	60	0.045	Contributes to cuticular wax and suberin biosynthesis. Involved in both decarbonylation and acyl-reduction wax synthesis pathways.
